# Maternal Vitamin D Status in the Late Second Trimester and the Risk of Severe Preeclampsia in Southeastern China

**DOI:** 10.3390/nu9020138

**Published:** 2017-02-14

**Authors:** Xin Zhao, Rui Fang, Renqiang Yu, Daozhen Chen, Jun Zhao, Jianping Xiao

**Affiliations:** 1 Department of Obstetrics, Wuxi Maternity and Child Health Hospital Affiliated to Nanjing Medical University, Wuxi 214002, China; zhaoxin63147@163.com; 2 Centre for Reproductive Medicine, Wuxi Maternity and Child Health Hospital Affiliated to Nanjing Medical University, Wuxi 214002, China; wfangruix@163.com; 3 Department of Newborn, Wuxi Maternity and Child Health Hospital Affiliated to Nanjing Medical University, Wuxi 214002, China; yurenqiang553@163.com; 4 Central Laboratory, Wuxi Maternity and Child Health Hospital Affiliated to Nanjing Medical University, Wuxi 214002, China; chendaozhen@163.com (D.C.); chalange@163.com (J.Z.)

**Keywords:** vitamin D status, serum 25(OH)D, pregnant women, severe preeclampsia

## Abstract

The association between maternal vitamin D deficiency and the risk of severe preeclampsia is still debated. In the present study, we aimed to evaluate vitamin D status in Chinese pregnant women and investigate its correlation with the odds of developing severe preeclampsia. A cohort study was performed on 13,806 pregnant women who routinely visited the antenatal care clinics and subsequently delivered at the Wuxi Maternity and Child Health Hospital. All the subjects in the cohort had their serum 25-hydroxyvitamin D (25(OH)D) concentrations measured during pregnancy. A high prevalence of maternal vitamin D deficiency (25(OH)D < 50 nmol/L) was found. Pregnant women who had different BMIs before pregnancy had significantly different serum concentrations of 25(OH)D. There was also a significant difference in the serum 25(OH)D concentration among pregnant women of different ages. The serum 25(OH)D concentration was significantly lower in pregnant women who subsequently developed severe preeclampsia compared with those who did not. Maternal vitamin D deficiency at 23–28 weeks of gestation was strongly associated with increased odds for severe preeclampsia after adjusting for relevant confounders (adjusted OR, 3.16; 95% CI, 1.77–5.65). Further studies are required to investigate whether vitamin D supplementation would reduce the risk of severe preeclampsia and improve pregnancy outcomes.

## 1. Introduction

Vitamin D is an important nutrient for human health [1]. As a steroid hormone precursor, it is primarily made in the skin through exposure to ultraviolet B radiation. Vitamin D undergoes hydroxylation to become 25-hydroxyvitamin D (25(OH)D) in the liver, which is subsequently converted to its active form 1,25-dihydroxyvitamin D (1,25(OH)_2_D) in the kidney, the placenta and other target organs [2]. A high prevalence of vitamin D deficiency has been reported worldwide, and increasing concerns have also been raised regarding maternal vitamin D deficiency and its detrimental impacts [3,4]. Vitamin D deficiency during pregnancy might cause multiple adverse health problems in mothers and infants, which include abnormal fetal bone metabolism and maternal postnatal depression that may persist into later life [5]. In addition to the classical effect on bone mineralization and calcium absorption, vitamin D plays a critical role in immunomodulation. It is suggested that vitamin D might help regulate maternal inflammatory responses to the fetus, which is very important in maintaining a normal pregnancy [6,7].

Preeclampsia is a multisystem pregnancy disorder defined by new-onset hypertension and proteinuria after 20 weeks of gestation [8,9]. Preeclampsia affects 2%–8% of all pregnancies and remains a leading cause of maternal and perinatal mortality and morbidity, including preterm birth and small for gestation age [10]. Delivery of the placenta is the only known effective cure for preeclampsia. Mothers with a history of preeclampsia are at increased risks for cardiovascular diseases later in life [11]. Severe preeclampsia occurs in 1%–2% of all pregnancies, which might be complicated with abruptio placentae, acute renal failure, or haemolysis, elevated liver enzymes, and low platelet (HELLP) syndrome, and it is often associated with higher risks of adverse pregnancy outcomes [11,12]. Although the diagnosis of preeclampsia is well characterized, the aetiology remains elusive. Clinical manifestations of preeclampsia normally appear in the second half of pregnancy, but the potential pathogenic mechanisms might occur much earlier. Preeclampsia is hypothesized to be associated with several contributing factors including abnormal trophoblast invasion, reduced placental perfusion, impaired spiral artery remodeling, endothelial dysfunction, oxidative stress and excessive maternal inflammatory responses [13,14].

Numerous potential nutritional targets for preeclampsia intervention have been suggested. There is a growing interest in the role of maternal vitamin D status in the pathophysiological development of preeclampsia. It has been reported that serum levels of inflammatory cytokines, such as IL-6, TNF-α and IL-10, are obviously elevated in women with preeclampsia [15,16]. Vitamin D has immunosuppressive effects, and vitamin D deficiency is reported to be associated with increased secretion of proinflammatory cytokines in healthy women [17]. In vitro studies have shown that 1,25(OH)_2_D_3_ could modulate IL-6 and TNF-α expression by suppressing NF-κB [18,19]. It is also found that vitamin D inhibits activation and proliferation of T cells and stimulates the IL-10 secretion and T-regulatory cell production, which are vital in maternal immune tolerance for normal placenta implantation [20,21].

Several epidemiological studies have investigated the relationship between maternal vitamin D status and preeclampsia [22,23], and an association between maternal vitamin D deficiency and severe preeclampsia has been reported [12,24]. However, findings are inconsistent and not all studies have detected this association [25,26,27]. Most of the studies are conducted predominantly in Caucasian populations, and some studies are limited by small sample sizes, and relevant confounding factors such as blood sampling season are not controlled for. In addition, there is no consensus on vitamin D supplementation guidelines for Chinese pregnant women due to insufficient data on vitamin D status for this population, and large-scale epidemiological studies on the association between vitamin D and adverse pregnancy outcomes as well as appropriate RCTs are also lacking. The objective of our study was to investigate the nutritional vitamin D status in Chinese pregnant women of a large cohort and determine the association between vitamin D deficiency at 23–28 weeks of gestation and the odds of severe preeclampsia.

## 2. Materials and Methods

### 2.1. Study Population

This study was performed on pregnant women who routinely visited the antenatal care clinic and subsequently delivered at the Wuxi Maternity and Child Health Hospital between January 2011 and December 2013. The Wuxi Maternity and Child Health Hospital is a tertiary health-care centre with approximately 12,000 deliveries per year, mainly providing service for the urban residents in Wuxi, south of Jiangsu province in China. All the participants in our study were from the Wuxi urban area (latitude 31.57°N) and those supplemented with calcium and vitamin D before blood sampling were excluded. Pregnant women with fetal anomalies or pre-existing medical conditions including chronic hypertension, renal disease and pregestational diabetes were also excluded. A total of 13,806 pregnant women were recruited in this study, and their non-fasting blood samples were banked during pregnancy. Among the subjects, 11,151 pregnant women gave their blood during 23–28 weeks of gestation and they were included in the further study investigating the association between maternal vitamin D and the risk of severe preeclampsia. Informed written consent was obtained from all the subjects. This study was conducted in accordance with the Declaration of Helsinki and was approved by the Medical Research Ethics Board of Wuxi Maternity and Child Health Hospital.

### 2.2. Definition of Severe Preeclampsia

We applied the international criteria to define preeclampsia as the presence of hypertension (systolic blood pressure (SBP) ≥140 mmHg and/or diastolic blood pressure (DBP) ≥90 mmHg) on 2 occasions at least 6 h apart after 20 weeks of gestation with detectable proteinuria of ≥0.3 g/24 h or >1 by urine dipstick in previously normotensive women [8,11,28]. According to the International Society for the Study of Hypertension in Pregnancy (ISSHP) criteria, severe preeclampsia was diagnosed if at least one of the following symptoms appeared: SBP ≥160 mmHg; DBP ≥110 mmHg; any evidence of other organ-damage including proteinuria of ≥3 g/24 h or 3+ on urine dipstick, oliguria, pulmonary edema, liver dysfunction, thrombocytopenia or central nervous system disturbance (altered vision, headache).

### 2.3. Data Collection

Demographic information including maternal age, gravidity, parity, pre-pregnancy body mass index (BMI) and season of conception was collected via in-person interviews at enrollment in the first trimester. Pre-pregnancy BMI (weight (kg)/height (m)^2^) was based on self-reported pre-pregnancy weight and measured height. If the pre-pregnancy weight was not known, the weight at the first visit in the first trimester was used as a proxy. Clinical data on gestational age, season at blood sampling and the serum level of total calcium were obtained from medical records. Gestational age was determined according to the date of the last menstrual period and was confirmed by ultrasound reports in the first trimester. Season of conception and season at blood sampling was classified as spring (from 19 March to 28 May), summer (from 29 May to 3 October), autumn (from 4 October to 30 November), and winter (from 1 December to 18 March) according to the distinct climatic features in Wuxi [4,29]. Medical records were abstracted to ascertain diagnosis of severe preeclampsia based upon blood pressure and urine protein measurements throughout gestation, antepartum and delivery events. Data on SBP, DBP and urine protein were also recorded. A delivery summary of gestational age, maternal BMI at delivery, birth weight and 1-min/5-min Apgar score was recorded by obstetricians.

### 2.4. Sample Collection and 25(OH)D Assay

Non-fasting maternal blood samples were collected and banked during pregnancy from 13,806 eligible pregnant women, and the majority of them (*n* = 11,151, 80.8%) were collected during the late midtrimester at 23–28 weeks of gestation when the routine antenatal screening for gestational diabetes mellitus was performed. Serum samples were immediately separated by centrifugation at 3500 rpm at 4 °C for 10 min and stored in aliquots at −80 °C. 25(OH)D is highly stable in the serum with a half-life of approximately 3 weeks and is thus considered as an accurate serum biomarker to indicate vitamin D status [30,31]. The serum 25(OH)D concentration was measured using an automated chemiluminescence immunoassay (Liaison; DiaSorin, Stillwater, MN, USA). The lower and upper limit of 25(OH)D detection was 10 nmol/L and 375 nmol/L, respectively. Two levels of internal quality control provided by the manufacturer (DiaSorin Inc., Stillwater, MN, USA) were run in duplicates in each assay. The inter-assay and intra-assay coefficients of variation were 5.2% and 8%, respectively. There is no universally accepted definition of maternal vitamin D deficiency during pregnancy, so we used the cut-off point suggested by the Endocrine Society, and vitamin D status was categorized as deficient when the serum 25(OH)D level was below 50 nmol/L [32]. A similar cut-off point for vitamin D deficiency had been used previously in the literature [4].

### 2.5. Statistical Analysis

All statistical analyses were performed using SPSS software version 20.0 (SPSS Inc., Chicago, IL, USA). Continuous variables were compared using the Mann–Whitney U-test or Kruskal–Wallis test, while categorical variables were compared using the Chi Square (χ^2^) test. The difference was considered as statistically significant when *p* < 0.05. Binary logistic regression analysis was used to determine the association between maternal serum 25(OH)D (<50 nmol/L versus ≥50 nmol/L) and the odds of severe preeclampsia. Unadjusted/adjusted odds ratios (ORs) and 95% CIs were calculated. The relevant confounding variables controlled for in the binary logistic regression analysis included pre-pregnancy BMI, maternal age, parity and season of maternal blood sampling, which were considered to be associated with the risk of severe preeclampsia or associated with vitamin D status.

## 3. Results

### 3.1. Distribution of Serum 25(OH)D Concentrations During Pregnancy

**Figure 1:**
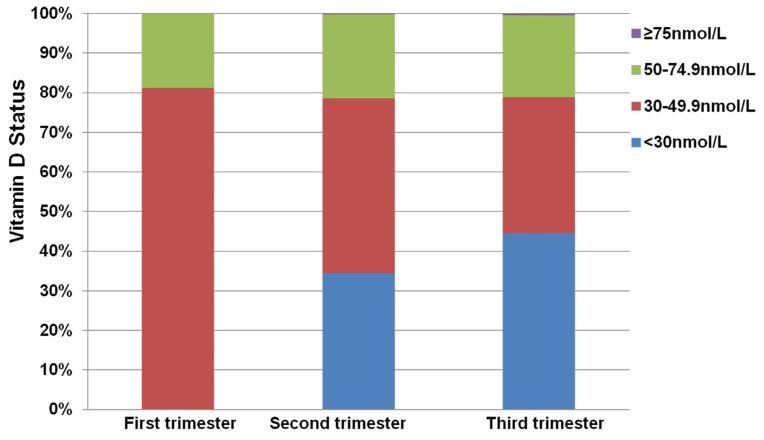
Vitamin D status among pregnant women in the first, the second and the third trimester. First trimester, 1–12 weeks of gestation (*n* = 38); second trimester, 13–28 weeks of gestation (*n* = 12,000); third trimester, 29–40 weeks of gestation (*n* = 1768). The vitamin D status was indicated as the mean value of the serum 25-hydroxyvitamin D (25(OH)D) level (<30 nmol/L; 30–49.9 nmol/L; 50–74.9 nmol/L and ≥75 nmol/L).

The maternal age of all subjects ranged from 17–42 years with a mean (SD) of 27.3 (3.9) years (*n* = 13,806). A total of 38, 12,000 and 1768 pregnant women had their serum 25(OH)D concentration measured in the first, the second and the third trimester, respectively. The percent of pregnant women with a serum 25(OH)D level below 50 nmol/L in the first, the second and the third trimester was 81.3%, 77.4% and 78.8%, respectively (Figure 1).

### 3.2. Maternal Vitamin D Status at 23–28 Weeks of Gestation

The mean value of the serum 25(OH)D concentration of 11,151 pregnant women measured at 23–28 weeks of gestation was 37.7 nmol/L (Table 1). We found a significant difference in the serum 25(OH)D concentration among pregnant women at different ages (*p* < 0.001), and the serum 25(OH)D concentration was obviously lower in pregnant women over 35 years. Based on their pre-pregnancy BMIs, pregnant women were categorized as underweight (<18 kg/m^2^), normal-weight (18–24.9 kg/m^2^) or overweight (≥25 kg/m^2^), and the serum 25(OH)D concentration was significantly different among them (*p* < 0.001). Pregnant women who were overweight before pregnancy had an obviously lower serum 25(OH)D, while there was no significant difference in the serum 25(OH)D concentration between pregnant women who were normal weight and underweight before pregnancy. No significant difference was observed in the midgestational serum 25(OH)D concentration among mothers of infants with low birth weight (<2500 g), normal birth weight (2500–4000 g) and high birth weight (>4000 g) (*p* = 0.344). The maternal 25(OH)D concentration was not significantly different between mothers with a preterm delivery (<37 week of gestation) and mothers with a term delivery (≥37 week) (*p* = 0.489). Multiparous women had a significantly higher serum concentration of 25(OH)D compared with nulliparous women (*p* < 0.001). Remarkable seasonal variations in the maternal 25(OH)D concentration were found (*p* < 0.001) and were highest when blood was drawn in summer (44.8 ± 15.2 nmol/L) and lowest in winter (30.6 ± 9.9 nmol/L).

**Figure 2:**
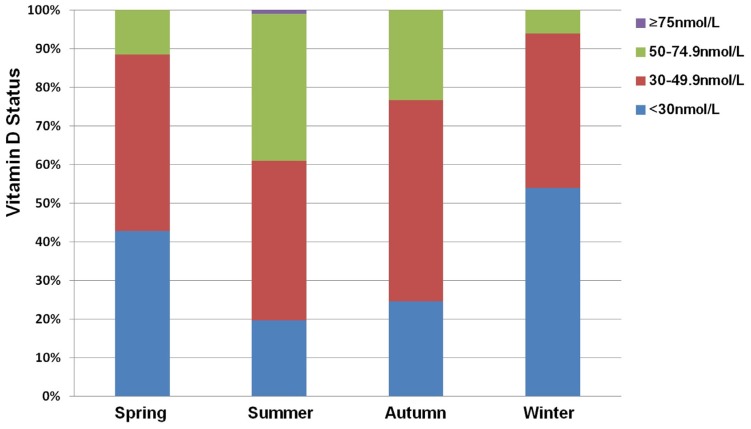
Vitamin D status among pregnant women based on different seasons for blood sampling. The vitamin D status was indicated as the serum 25-hydroxyvitamin D (25(OH)D) level (<30 nmol/L; 30–49.9 nmol/L; 50–74.9 nmol/L and ≥75 nmol/L). Season of blood sampling was classified as spring (from 19 March to 28 May), summer (from 29 May to 3 October), autumn (from 4 October to 30 November), and winter (from 1 December to 18 March). The prevalence of maternal vitamin D deficiency (25(OH)D < 50 nmol/L) was highest in winter (93.9%) and lowest in summer (60.9%).

In this cohort, the prevalence of vitamin D deficiency (<50 nmol/L) was 78.9% (Table 2) and only 0.3% of women had a serum 25(OH) D level over 75 nmol/L. Vitamin D deficiency was most common (91.4%) in pregnant women over 35 years. The prevalence of vitamin D deficiency was significantly higher in pregnant women who had a BMI ≥25 kg/m^2^ before pregnancy (*p* < 0.001). However, there was no significant difference in the prevalence of vitamin D deficiency between mothers with a preterm delivery and mothers with a term delivery (*p* = 0.05), or between multiparous and nulliparous pregnant women (*p* = 0.601). A serum 25(OH)D below 50 nmol/L was most common in winter (93.9%) and least common in summer (60.9%) (Figure 2).

### 3.3. Vitamin D Status and the Risk of Severe Preeclampsia

Among 11,151 pregnant women whose vitamin D status was assessed during 23–28 weeks of gestation, 139 (1.2%) subsequently developed severe preeclampsia. As expected, there was a significant difference in SBP, DBP, urine protein per 24 h, gestational age, maternal BMI at delivery, 1-min/5-min Apgar score and birth weight between severe preeclampsia cases and control cases (*p* < 0.001) (Table 3). The midgestational serum 25(OH)D concentration was found to be significantly lower in women with severe preeclampsia compared with those without (*p* < 0.001), while no significant difference was found in the serum concentration of total calcium between severe preeclampsia cases and controls (*p* = 0.129). The other demographic and clinical characteristics of pregnant women developing severe preeclampsia are described in Table 3. There was a significant difference in the incidence of severe preeclampsia in pregnant women with different vitamin D status (<50 versus ≥50 nmol/L, *p* = 0.002) (Table 4). A total of 1.4% of pregnant women with vitamin D deficiency (<50 nmol/L) subsequently developed severe preeclampsia, while only 0.6% of pregnant women with sufficient vitamin D (≥ 50 nmol/L) developed severe preeclampsia. Binary logistic regression analysis indicated that vitamin D deficiency was a high risk factor for severe preeclampsia (OR: 2.20; 95% CI: 1.31–3.72) (Table 5). After controlling for confounding factors including maternal age, parity, pre-pregnancy BMI and season of maternal blood sampling, a more than 3-fold increase in the odds of severe preeclampsia was observed in pregnant women with vitamin D deficiency (adjusted OR: 3.16; 95% CI: 1.77–5.65).

## 4. Discussion

In the present study, we found a poor vitamin D status of pregnant women in Wuxi, southeastern China (31.5°N). Our investigation was focused on the maternal vitamin D status at 23–28 weeks of gestation. The prevalence of vitamin D deficiency (<50 nmol/L) was found to be high during midgestation. A previous study conducted in Nanjing (31°N), adjacent to Wuxi, found that the prevalence of maternal 25(OH)D level below 50 nmol/L at 24–28 weeks of gestation was 94.7% in summer and 96.1% in winter [33]. Studies on maternal vitamin D status during pregnancy in other regions of China have also been reported [34]. The prevalence of maternal vitamin D deficiency was 90.2% in Beijing, northern China (39.9°N) [35] and was 83.6% in Guiyang, southwestern China (27.2°N) [36]. In contrast, the percent of vitamin D-deficient pregnant women was much lower (18.9%) in Guangzhou, southern China (23°N) [37]. This difference might be attributed to covariates including geographic location, dietary vitamin D intake and lifestyles. The main vitamin D dietary sources in China are oily fish and fortified milk. A high prevalence of hypovitaminosis D (<50 nmol/L) was also found in pregnant Korean women (77.3%) [38] and pregnant Japanese women (89.5%) [39]. All these findings, as well as ours, suggest that vitamin D deficiency is common in pregnant women in most of the regions of East Asia.

It is well known that vitamin D_3_ production is affected by sunlight exposure, skin pigmentation and clothing habit. The majority of young women in East Asia spend limited time outdoors because of the cultural reason that they prefer fair skin to tanned skin. In addition to limited sunlight exposure, inadequate oral intake of vitamin D supplements is also a leading cause of vitamin D deficiency in Chinese pregnant women. A recent randomized controlled trial (RCT) study showed that vitamin D supplementation of 4000 IU/day for pregnant women starting at 12–16 weeks of gestation is both safe and effective in achieving vitamin D sufficiency (>50 nmol/L) throughout pregnancy [40]. The current recommendation of vitamin D intake by the Institute of Medicine is 400–600 IU/day [31,41], while the Endocrine Society recommends that pregnant women should take vitamin D supplements of 1500–2000 IU/day [32]. However, official guidelines for vitamin D supplementation are lacking in China.

Our study also demonstrated that the serum 25(OH)D concentration was much lower in pregnant women with a pre-pregnancy BMI of ≥25 kg/m^2^. It has been reported that being overweight or obese might have an adverse effect on nutritional vitamin D status [42]. Vitamin D is a group of fat-soluble prohormones, and the precursor of vitamin D is stored in adipose tissue, which might hinder conversion of the inactive vitamin D precursor into the bioactive form calcitriol [43,44]. Thus, it is suggested that pre-pregnancy weight control is important and beneficial for maternal vitamin D status during pregnancy.

Although several observational studies have described the relationship between maternal vitamin D status and the risk of preeclampsia, the findings are inconsistent. Bodnar first suggested that maternal vitamin D deficiency at <22 weeks of gestation was a strong independent risk factor for preeclampsia [23]. A recent meta-analysis including eight relevant studies has shown that vitamin D deficiency during pregnancy remarkably increases the odds of preeclampsia [45]. Our findings add to the evidence that maternal vitamin D deficiency at 23–28 weeks of gestation was associated with severe preeclampsia, and more than 3-fold increased odds of severe preeclampsia was observed among pregnant women with vitamin D deficiency after adjusting for confounders. A longitudinal study demonstrated similar findings, i.e., that low maternal serum 25(OH)D levels at 24–26 weeks of gestation were associated with an increased risk of preeclampsia [22]. Robinson published a case-control study that clearly presented a significantly lower serum 25(OH)D concentration among patients with early-onset severe preeclampsia [46]. Another nested case-control study reported by Baker also found an association between maternal vitamin D deficiency in the late mid-trimester and an increased risk of severe preeclampsia [24], which was in accordance with our study. A large prospective study involving 23,423 nulliparous women from Norway further supported the hypothesis that vitamin D supplementation might reduce the risk of preeclampsia [47]. However, Wetta failed to detect any association between maternal 25(OH)D concentrations at midgestation and the risk of preeclampsia [27]. Another study on women at high risks of preeclampsia also found no association between low serum levels of 25(OH)D before 20 weeks of gestation and the subsequent development of preeclampsia [26].

There are several potential reasons that might explain the dissimilarity in the findings of different studies. The main differences are the sample size and population characteristics including genetic background, the occurrence of preeclampsia and the unmeasured lifestyles [25]. Season and gestational age at the serum 25(OH)D measurement might also contribute to the differences, since seasonal variation in the incidence of preeclampsia has been reported, i.e., summer has the lowest risk of preeclampsia when sunlight is plentiful and vitamin D synthesis is most effective, while winter has the highest incidence of preeclampsia [47,48]. In our study, we collected blood samples equally across the four seasons, and we found that season of maternal blood sampling was associated with the odds of severe preeclampsia after adjusting for maternal vitamin D status (data not shown). Some of the previous studies separated cases by severity of preeclampsia, which is more convincing when the role of vitamin D in the pathogenesis of preeclampsia is discussed [12,26]. An important feature of our study was that we focused on severe preeclampsia cases. Bodnar has demonstrated that maternal vitamin D deficiency may be a risk factor for severe preeclampsia, but no association was found for overall preeclampsia or mild preeclampsia [12]. Future studies on large cohorts with strict definitions of preeclampsia separating cases into clinical subtypes are required to advance the research in this field.

There are multiple biologically plausible mechanisms underlying the contribution of maternal vitamin D deficiency to the pathophysiology of preeclampsia. Abnormal placental implantation, vascular endothelial dysfunction and excessive inflammation at the maternal-fetal interface characterize preeclampsia [49]. Preeclampsia is hypothesized to be a two-stage pregnancy disorder. Stage I involves reduced placental perfusion secondary to abnormal implantation [50]. Vitamin D plays a role in early placental development via regulation of implantation-related genes, and the metabolism of vitamin D in placenta is found to be altered in preeclampsia cases, which might lead to abnormal trophoblastic invasion in stage I of preeclampsia [51]. The poor perfused placenta produces molecules initiating the ensuing multisystem sequelae, which are proposed to be secondary to endothelial dysfunction as stage II of preeclampsia [52].

As the bioactive form of vitamin D, 1,25(OH)_2_D_3_ has immunomodulatory properties and regulates angiogenic processes by promoting secretion of vascular endothelial growth factor and expression of antioxidant CuZn superoxide dismutase through vitamin D receptor (VDR)-mediated pathways [53]. In vitro studies have shown that 1,25(OH)_2_D_3_ can regulate endothelial progenitor cell migration and angiogenesis [54]. NF-κB is involved in the transcription of multiple proinflammatory cytokines, and it also plays an important role in immune responses of T helper 1 and T helper 2 cytokines. It has been found that NF-κB activity in activated peripheral blood lymphocytes and in placenta is much higher among preeclamptic patients compared with healthy controls [55]. It is demonstrated that VDR interacts with IκB kinase β to block NF-κB activation and reduce the expression of proinflammatory factors. 1,25(OH)_2_D_3_ is found to up-regulate VDR inhibition of NF-κB activation in a dose-dependent manner [56]. Vitamin D deficiency during pregnancy might contribute to an inappropriate NF-κB activation in placental tissue, leading to over-expression of proinflammatory cytokines and excessive inflammation at the maternal-fetal interface, which might partly explain the increased risk of preeclampsia among vitamin D-deficient pregnant women. In observational studies performed on non-pregnant women, vitamin D deficiency is found to be associated with hypertension [57]. Animal studies have shown that vitamin D deficiency correlates with cardiovascular dysfunction including cardiac hypertension and hypertrophy [57].

China, as the largest developing country in the world, still has large differences in the economic condition, nutritional status and lifestyles of citizens compared with developed countries. Studies regarding the association between maternal vitamin D status and the risk of severe preeclampsia are very limited in developing countries. Our study does have several strengths including its large sample size of Chinese pregnant women with a wide range in maternal age, parity, pre-pregnancy BMI and blood sampling season. The ethnically homogeneous population of pregnant women in this study was also a major strength. We followed up pregnancy outcomes of participants and compared the midgestation vitamin D status at 23–28 weeks of gestation in severe preeclampsia cases with normal pregnancy cases. This gestational age range (23–28 weeks) allowed us to capture severe preeclampsia cases and to assess maternal vitamin D status before the clinical onset of most cases. In addition, we excluded pregnant women with fetal anomalies or pre-existing chronic medical conditions such as chronic hypertension and pregestational diabetes, which have been suggested to be associated with increased risk of preeclampsia.

Our study also has several limitations that should be addressed in future investigations. Sociodemographic information and data on dietary structure, lifestyles and household income of participants were not collected. The baseline dietary intake of vitamin D was not assessed. Maternal serum 25(OH)D concentrations were not longitudinally measured throughout gestation. In addition, unmeasured confounding factors may exist. A causal relationship between vitamin D deficiency and severe preeclampsia cannot be determined.

## 5. Conclusions

Vitamin D deficiency at midgestation is common among Chinese pregnant women. Pre-pregnancy overweight and obesity had an adverse effect on maternal vitamin D status during pregnancy. The serum 25(OH)D concentration was much lower and the prevalence of vitamin D deficiency was much higher in pregnant women aged over 35 years. Vitamin D deficiency at 23–28 weeks of gestation is associated with an increased risk of the development of severe preeclampsia. Further studies are required to elucidate whether adequate vitamin D supplementation for Chinese women in the preconception period or in early pregnancy would reduce the risk of severe preeclampsia. Stronger evidence from epidemiologic studies with larger sample sizes controlling for important covariates is needed to confirm our findings. Additional well-designed and randomized trials of vitamin D supplementation are necessary to determine the role of vitamin D and establish guidelines for vitamin D intake for the prevention of severe preeclampsia.

## Figures and Tables

**Table 1:** The serum 25(OH)D concentration of pregnant women stratified by different characteristics.

Characteristics	*n* (%)	Serum 25(OH)D (nmol/L)		*p* Value
		Mean (Range) SD		
**Total**	11,151 (100)	37.7 (10.0–76.7)	14.1	
**Age**				<0.001
17–24 (year)	2960 (26.5)	37.8 (10.1–76.6)	14.1	
25–29 (year)	5298 (47.5)	38.6 (10.0–76.7)	14.5	
30–34 (year)	2226 (20.0)	36.5 (10.0–76.5)	13.4	
≥35 (year)	667 (6.0)	33.7 (11.4–70.3)	11.8	
**Pre-pregnancy BMI**				<0.001
<18 kg/m^2^	768 (6.9)	38.4 (11.0–74.0)	14.8	
18–24.9 kg/m^2^	9353 (83.9)	38.0 (10.0–76.6)	14.1	
≥25 kg/m^2^	1030 (9.2)	34.5 (10.0–70.0)	12.7	
**BW**				0.344
<2500 g	255 (2.3)	38.7 (10.1–73.9)	16.2	
2500–4000 g	10,436 (93.6)	37.6 (10.0–76.6)	14.0	
>4000 g	460 (4.1)	38.2 (13.1–72.9)	14.4	
**GA at delivery**				0.489
<37 week	474 (4.3)	38.2 (10.1–74.0)	15.3	
≥37 week	10,677 (95.7)	37.7 (10.0–76.6)	14.0	
**Parity**				<0.001
nulliparous	9918 (88.9)	37.5 (10.0–76.6)	14.1	
multiparous	1233 (11.1)	39.0 (13.6–76.3)	13.7	
**Season of blood sampling**				<0.001
Spring	2059 (18.5)	33.8 (14.5–65.7)	12.3	
Summer	3390 (30.4)	44.8 (10.0–76.6)	15.2	
Autumn	2535 (22.7)	40.2 (10.0–72.3)	12.8	
Winter	3167 (28.4)	30.6 (10.1–56.0)	9.9	

25(OH)D, 25-hydroxyvitamin D; BMI, body mass index; BW, birth weight; GA, gestational age. The comparison was made using the Mann–Whitney U test.

**Table 2:** The serum 25(OH)D concentration of pregnant women according to categories of relevant confounding variables.

Category	*n*	Serum 25(OH)D (nmol/L)			*p* Value
		<50 *n* (%)	50–74.9 *n* (%)	≥75 *n* (%)	
**All**	11,151	8799 (78.9)	2318 (20.8)	34 (0.3)	
**Age**					<0.001
17–24 (year)	2960	2317 (78.3)	637 (21.5)	6 (0.2)	
25–29 (year)	5298	4019 (75.8)	1255 (23.7)	24 (0.5)	
30–34 (year)	2226	1878 (84.3)	344 (15.5)	4 (0.2)	
≥35 (year)	667	610 (91.4)	57 (8.6)	0	
**Pre-Pregnancy BMI**					<0.001
<18 kg/m^2^	768	585 (76.2)	183 (23.8)	0	
18–24.9 kg/m^2^	9353	7305 (78.1)	2011 (21.5)	37 (0.4)	
≥25 kg/m^2^	1030	609 (88.3)	121 (11.7)	0	
**GA at delivery**					0.05
<37 weeks	474	357 (75.3)	117 (24.7)	0	
≥37 weeks	10,677	8443 (79.1)	2200 (20.6)	34 (0.3)	
**Parity**					0.601
nulliparous	9918	7830 (78.9)	2058 (20.8)	30 (0.3)	
multiparous	1233	966 (78.4)	258 (20.9)	9 (0.7)	

25(OH)D, 25-hydroxyvitamin D; BMI, body mass index; BW, birth weight; GA, gestational age. The comparison was made using the χ^2^ test.

**Table 3:** Demographic and clinical characteristics of pregnant women with severe preeclampsia and those without.

Characteristics	SPE Case *n* = 139	Non-SPE Case *n* = 11,012	*p* Value
**Age (year)**	27.3 ± 4.3	27.3 ± 3.9	0.605
**Pre-Pregnancy BMI (kg/m^2^)**	21.7 ± 3.5	21.4 ± 2.6	0.945
**Gravidity**	1.9 ± 1.1	2.0 ± 1.1	0.951
**Parity**	1.1 ± 0.3	1.1 ± 0.3	0.644
**Season of conception**			<0.001
Spring	17 (12.2)	2748 (24.9)	
Summer	61 (43.9)	3269 (29.7)	
Autumn	12 (8.6)	2048 (18.6)	
Winter	49 9 (35.3)	2947 (26.8)	
**Season of blood sampling**			<0.001
Spring	29 (20.9)	2030 (18.4)	
Summer	48 (34.5)	3342 (30.4)	
Autumn	13 (9.4)	2522 (22.9)	
Winter	49 (35.2)	3118 (28.3)	
**Calcium (mmol/L)**	1.6 ± 0.1	1.7 ± 0.3	0.01
**25(OH)D (nmol/L)**	32.8 ± 11.7	37.7 ± 14.1	<0.001
**SBP (mmHG)**	154.0 ± 8.4	121.5 ± 8.2	<0.001
**DBP (mmHG)**	107.0 ± 10.3	77.0 ± 6.5	<0.001
**Urine protein (g/24 h)**	2.7 ± 0.7	0.0 ± 0.1	<0.001
**BW (g)**	2689 ± 835	3358 ± 935	<0.001
**BMI at delivery (kg/m^2^)**	28.4 ± 4.5	27.3 ± 3.0	<0.001
**GA at delivery (week)**	36.1 ± 3.2	39.0 ± 1.4	<0.001
**1-min Apgar score**	8.6 ± 2.8	9.9 ± 0.4	<0.001
**5-min Apgar score**	9.14 ± 2.1	10.0 ± 0.2	<0.001

SPE, severe preeclampsia; BMI, body mass index; 25(OH)D, 25-hydroxyvitamin D; SBP, systolic blood pressure; DBP, diastolic blood pressure; BW, birth weight; GA, gestational age. Data are presented as the mean ± SD or number (percentage). The comparison between severe preeclampsia cases and control cases was made using the Mann–Whitney U test or χ^2^ test.

**Table 4:** Incidence of severe preeclampsia according to maternal serum 25(OH)D concentration at 23–28 weeks of gestation.

Maternal Serum 25(OH)D	Case		*p* Value
	SPE (*n* = 139)	Non-SPE (*n* = 11,012)	
<50 nmol/L (*n* = 8682)	123 (1.4)	8559 (98.6)	0.002
≥50 nmol/L (*n* = 2469)	16 (0.6)	2453 (99.4)	

25(OH)D, 25-hydroxyvitamin D; SPE, severe preeclampsia. Data are presented as a number (percentage). The comparison in the rate of severe preeclampsia was made using the χ^2^ test.

**Table 5:** Unadjusted and adjusted ORs for severe preeclampsia according to maternal vitamin D at 23–28 weeks of gestation.

Maternal 25(OH)D	SPE (*n*)	Non-SPE (*n*)	Unadjusted OR (95% CI)	*p* Value	Adjusted OR * (95% CI)	*p* Value
**<50 nmol/L**	123	8559	2.20 (1.31–3.72)	0.003	3.16 (1.77–5.65)	0.000
**≥50 nmol/L**	16	2453	1.00 (Reference)		1.00 (Reference)	

25(OH)D, 25-hydroxyvitamin D; SPE, severe preeclampsia; OR, odds ratio; CI, confidence interval; * Adjusted for pre-pregnancy BMI, maternal age, parity and season of maternal blood sampling.
